# Selection of best videos of the year for 2022

**DOI:** 10.1590/S1677-5538.IBJU.2023.01.03

**Published:** 2023-01-26

**Affiliations:** 1 Department of GU Oncology Moffitt Cancer Center FL USA Department of GU Oncology Moffitt Cancer Center, FL, USA; 2 Department of Tumor Biology Moffitt Cancer Center FL USA Department of Tumor Biology Moffitt Cancer Center, FL, USA

## COMMENT

Dear readers,

It is a distinct pleasure to once again highlight the significant contributions made to the International Brazilian Journal of Urology over the past year. Our journal has continued to expand its global reputation and broad readership over a tumultuous period of a global pandemic and diverse world conflicts as well as challenges. It is a true testament to the commitment of our journal and contributors to forge through in advancing science and surgical innovation during these times, with a direct consequence of this being an increasing recognition of our journal among the top tiered urological journals which is so very well depicted by our rapidly rising impact factor now benchmarked with many of the very best in our field.

In making the selection of best videos of the year, several criteria have been employed including their surgical novelty and potential to improve patient care. These are clearly high bars set but truthfully I am thrilled to report that these challenging criteria have been achieved to a large extent by many of our published videos so all contributing authors of accepted videos over the past year are to be congratulated nevertheless there can only be a few select winners and on that note, I am pleased to announce that the first prize for best video of the year is awarded to the submission by Moschovas MC et al. entitled “Da Vinci SP radical prostatectomy: A multicentric collaboration and step-by-step techniques” published in the July-August issue (Vol 48(4):728-729) of our journal (1). The video provides a very practical approach to conducting minimally invasive radical prostatectomy using a single port platform as defined by some of the premier global surgical leaders in this milieu. The authors are applauded for sharing their wealth of knowledge and technical refinements in making this surgical approach highly accessible to many surgeons seeking to offer this surgical modality within their patient population. The second prize is awarded to Inzillo R et al. from the department of Urology from Guastalla Hospital from Emilia-Romagna, Italy for their video entitled “Percutaneous and endoscopic combined treatment of bladder and renal lithiasis in Mitrofanoff conduit” which was published in our May-June issue (Vol48(3)598-599) (2). As nicely detailed by the authors, the significant benefits to adopting a purely endoscopic management in managing bladder and renal calculi in patients with a Mitrofanoff conduit cannot be overemphasized. A very well strategized and performed surgical approach is demonstrated which can serve as a framework for colleagues managing such patients to consider and potentially adopt, with remarkable benefits offered to patients in doing so in terms of quicker recovery and decreased potential surgical morbidity. The third prize is awarded to the video publication by Grosso AA et al. entitled “Three-dimensional reconstruction and intraoperative ultrasonography: Crucial tools to safely approach highly complex renal masses” from the department of experimental and clinical medicine, University of Florence in Italy which was published in our November-December issue (Vol 48(6):996-997) (3). This video is a beautiful depiction on how novel imaging tools (both pre- and intra-operative) can be employed and integrated as powerful tools to not only guide but refine our surgical approach to highly complex renal masses being managed using minimally invasive techniques in the completion of nephron sparing surgery. This is becoming an increasingly frequent surgical case type completed by many urologists and urologic oncologists throughout the world hence the implications of this work are significant with this video providing a nice overview on how this can be done seamlessly in day-to-day practice.

In my closing remarks, I would like to thank our dedicated readers and submitting contributors in allowing our journal to achieving new echelons of global recognition within the urological community and we are very excited in envisioning the promising future ahead.

Warmest regards and best wishes,
